# 9.H. Workshop: Understanding the institutional context of health inequalities. A life stage approach

**DOI:** 10.1093/eurpub/ckac129.579

**Published:** 2022-10-25

**Authors:** Organised by: Research Unit FOR2723(Germany), Chair persons: Irene Moor (Germany), Dennis Jepsen (Germany)

## Abstract

**Background:**

The health of young people is strongly linked to their socioeconomic background. However, there is little systematic research on the complex mechanisms underlying the association between family socioeconomic position and health among young people, particularly regarding contextual factors at the (intermediate) meso-level. Several institutional contexts are relevant for young people's lives: the family, kindergarten, primary and secondary school, higher education system, vocational school and training, workplace and the healthcare system. Previous studies demonstrated that characteristics of these institutional contexts are associated with young people's health and well-being. It is unclear, which role they play regarding health inequalities. The aim of the workshop is to analyse the role of institutional characteristics, such as compositional (which people are found in an institution) and contextual (the structural characteristics of an institution) factors for the development or existing health inequalities.

**Methods:**

The research unit FOR2723 analyses the role of meso-level, institutional characteristics regarding health inequalities considering the whole period of early life based a common conceptual framework. The results are based on empirical analyses of various secondary data, such as the National Educational Panel Study (NEPS) or the German Health Interview and Examination Survey for Children and Adolescents (KiGGS). Bodymass index (BMI) and the subjective self-assessment of health are considered as outcomes.

**Results:**

We found substantial social inequalities in health and health behaviour at any life stage investigated. In each life stage we found meso-level characteristics of the family (family investment, family stress, family atmosphere, and parental health behaviour and well-being), the kindergarten (the association between ECEC centre focus and family SEP on pre-school children's BMI), the school (composition of the school in terms of education, income, and occupational prestige of the students’ parents), the institutions and labour market states involved in the school-to-work transition (vocational training places, universities, workplaces, unemployment spells) as well as the health care system (regional health policy), which are associated with social inequalities in young people's self-rated health, subjective well-being and BMI.

**Discussion:**

In addition to micro-level determinants, meso-level characteristics of important institutions that young people are exposed to (family, kindergarten, school, school-to-work-transitions, healthcare system) are relevant for their health and reinforce health inequalities. More research is needed to understand how institutional contexts contribute to health inequalities and which institutional changes are needed to reduce inequalities.

**Key messages:**

• The role of institutional characteristics, such as compositional and contextual factors for health inequalities is unclear.

• Meso-level characteristics of institutions in young people (family, kindergarten, school, school-to-work-transitions, healthcare system) are relevant for their health and reinforce health inequalities.

